# The predictive value of systemic inflammatory markers in 902 patients with tunneled hemodialysis catheter

**DOI:** 10.1007/s40620-023-01880-w

**Published:** 2024-03-21

**Authors:** Melis Baykara Ulusan, Emine Meltem, Ilhan Nahit Mutlu, Kivilcim Ulusan

**Affiliations:** 1 Department of Diagnostic and Interventional Radiology, Istanbul Training and Research Hospital, Samatya, Istanbul, Turkey; 2 Department of Radiology, Department of Diagnostic and Interventional Radiology, Basaksehir Cam and Sakura City Hospital, Basaksehir, Istanbul, Turkey; 3 Department of General Surgery, Department of Endocrine Surgery, Istanbul Training and Research Hospital, Samatya, Istanbul, Turkey

**Keywords:** Hemodialysis, Tunneled hemodialysis catheter, Neutrophil–lymphocyte ratio, Platelet-lymphocyte ratio, Lymphocyte-monocyte ratio, Mean platelet volume

## Abstract

**Aim:**

This study aimed to assess the predictive role of neutrophil-to-lymphocyte ratio, platelet-to-lymphocyte ratio, lymphocyte-to-monocyte ratio, and mean platelet volume, on catheter survival in chronic hemodialysis patients, analyzing both infectious and non-infectious complications.

**Methods:**

A retrospective analysis encompassed 1279 tunneled catheter insertion procedures involving 902 patients between March 2014 and October 2018. Patients were categorized into two main groups: (i) initial placement and (ii) exchange. The exchange group was further stratified into four subgroups: infection, dysfunction, displacement, and transitioning temporary hemodialysis catheters to long-term ones. Hematologic ratios were calculated from baseline hemogram data, including neutrophil, lymphocyte, monocyte, and platelet counts, while mean platelet volume was derived from the same hemogram.

**Results:**

The patients in the exchange group displayed significantly higher lymphocyte and monocyte values (*p* < 0.001), while lower values were noted for neutrophil–lymphocyte ratio and platelet-lymphocyte ratio (*p* < 0.001). The transition group displayed higher monocyte values and lower mean platelet volume and lymphocyte-monocyte ratio values (*p* < 0.05). In the infection-related exchange subgroup, higher neutrophil count, mean platelet volume, neutrophil–lymphocyte ratio, and platelet-lymphocyte ratio values were observed compared to other groups (*p* < 0.05). Cases related to catheter dysfunction exhibited increased lymphocyte-monocyte ratio but lower neutrophil, monocyte, neutrophil–lymphocyte ratio, and platelet-lymphocyte ratio values (*p* < 0.05).

**Conclusion:**

This study highlights the interest of specific inflammatory markers, particularly monocytes, neutrophil–lymphocyte ratio, and platelet-lymphocyte ratio, in the management of tunneled catheters, notably in patients undergoing exchanges. However, cut-off values, essential for constructing management algorithms, are currently lacking, and prospective multicenter studies are needed for further elucidation.

**Graphical abstract:**

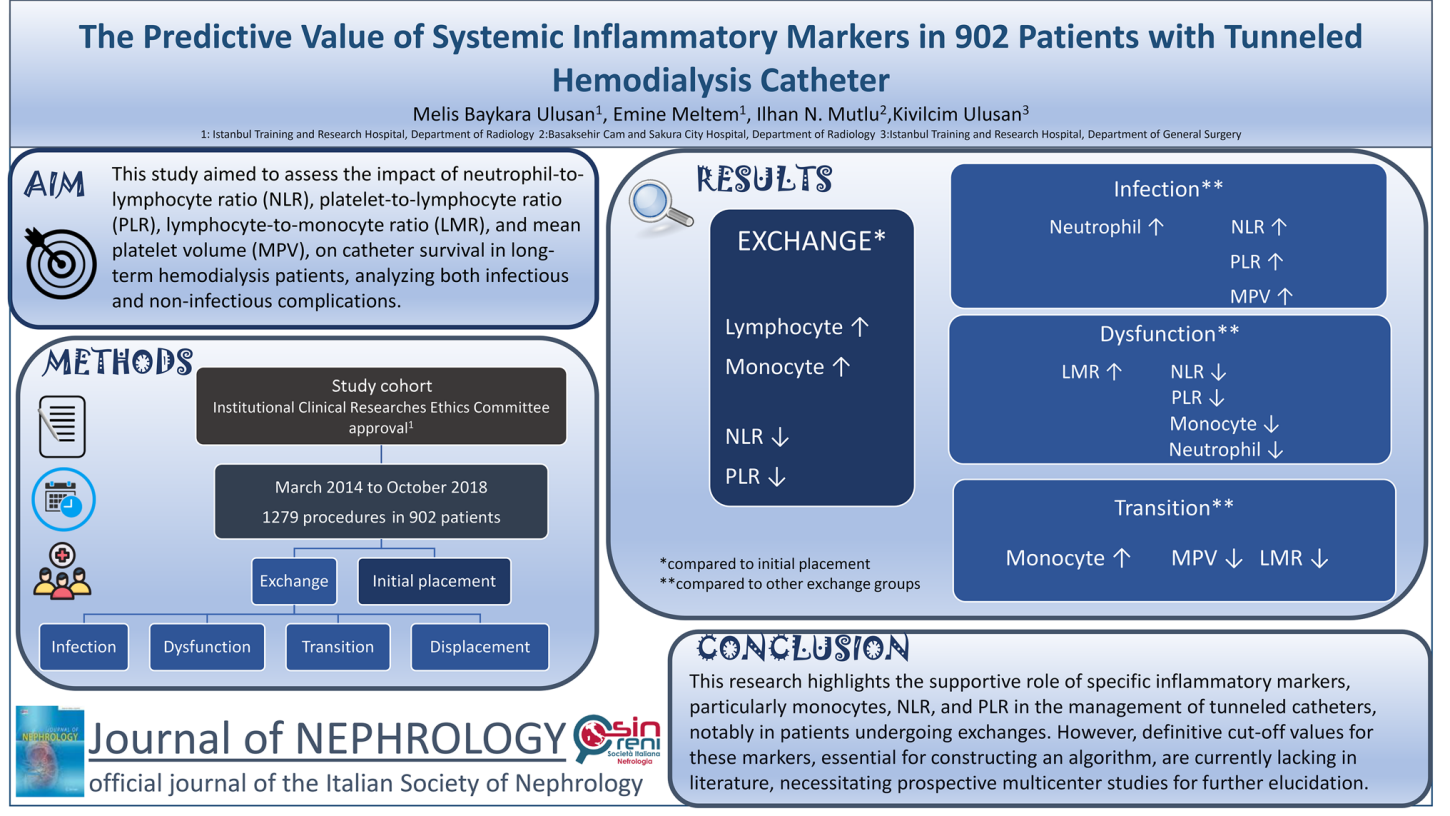

**Supplementary Information:**

The online version contains supplementary material available at 10.1007/s40620-023-01880-w.

## Introduction

Tunneled catheters represent a frequently used vascular access, often easing the lives of hemodialysis patients. They are commonly chosen for various reasons: in older patients with multiple health issues, or due to poor vascular patrimony with high chances of failure. In some cases, they are the final resort for hemodialysis when other access methods have been exhausted [1, 2].

The insertion of tunneled hemodialysis catheters allows a prompt and cost-effective means for immediate vascular access. Nevertheless, it comes with drawbacks, potentially leading to considerable complications such as infections, thrombosis, and dysfunction [3]. Tunneled catheters pose both infectious and non-infectious challenges that can lead to morbidity and mortality in individuals undergoing hemodialysis. Non-infectious issues encompass problems like catheter dysfunction, thrombosis, and central vein stenosis [4].

Infections are frequent complications in patients undergoing chronic hemodialysis. The risk of hospitalization due to infection and mortality are 2 to 3 times higher when compared to patients using arteriovenous fistulas or grafts. Several types of infections are associated with tunneled catheters, including catheter-related bloodstream infections, exit-site infections, and infections within the tunnel itself [5]. Conventional inflammatory indicators like C-reactive protein, procalcitonin, and ferritin are widely used in managing patients with end-stage kidney disease, but their sensitivity and specificity is too low to give guidance. Recent research has highlighted the correlation between the neutrophil-to-lymphocyte ratio, platelet-to-lymphocyte ratio, and inflammation in patients on chronic dialysis [6–8].

As previously mentioned, managing complications associated with catheters and their treatment presents a substantial financial strain in terms of healthcare expenses. In European countries, the cost for a patient experiencing a catheter-related bloodstream infection was assessed as being 29,909 € [9].

In our study, we investigated the relationship between catheter dysfunction and various blood parameters, seeking to fill gaps in current research by analyzing the neutrophil–lymphocyte ratio, platelet-lymphocyte ratio, lymphocyte-monocyte ratio, and mean platelet volume. Our focus was on understanding how these factors relate to catheter survival and to infectious and non-infectious complications in hemodialysis patients [10].

## Materials and methods

### Study design

We retrospectively retrieved data on 1279 tunneled catheter insertion procedures performed on 902 hemodialysis patients from March 2014 to October 2018.

The inclusion criteria were patients aged 18 to 90 who underwent catheter procedures at our interventional radiology clinic and standard thrice-weekly hemodialysis, and did not meet any of the exclusion criteria.

Exclusion criteria encompassed patients with a history of malignancy, hematological disease, rheumatological diseases, vasculitis, non-catheter related active infections (e.g., pneumonia, urinary tract infection), or those on immunosuppressants. These conditions were excluded due to their potential influence on the laboratory parameters investigated in this study. Patients lacking routine hemogram measurements within 24 h before the procedure were also excluded.

We collected data on patients’ demographics, clinical characteristics, treatment history, co-morbidities, site of catheter insertion, catheter survival, reason for catheter removal and incidence of catheter-related infection, laboratory data, procedure date, and type.

As routine, standard practice in our clinic, blood samples were collected from patients in the early morning after 8 h overnight fasting, 24 h before the catheter insertion/exchange procedure. Hemogram analyses were done by automated and standardized methods using Sysmex XN9000 (Sysmex America, Lincolnshire, IL).

Neutrophil–lymphocyte ratio, platelet-lymphocyte ratio, and lymphocyte-monocyte ratio were calculated from neutrophil, lymphocyte, monocyte, and platelet counts that were obtained from the baseline hemogram. The neutrophil–lymphocyte ratio and platelet-lymphocyte ratio were calculated as the ratio of neutrophils and platelets, respectively, to lymphocytes, while the lymphocyte-monocyte ratio was calculated as the ratio of absolute lymphocyte count to absolute monocyte count. The mean platelet volume was obtained from the baseline hemogram.

**Fig. 1:**
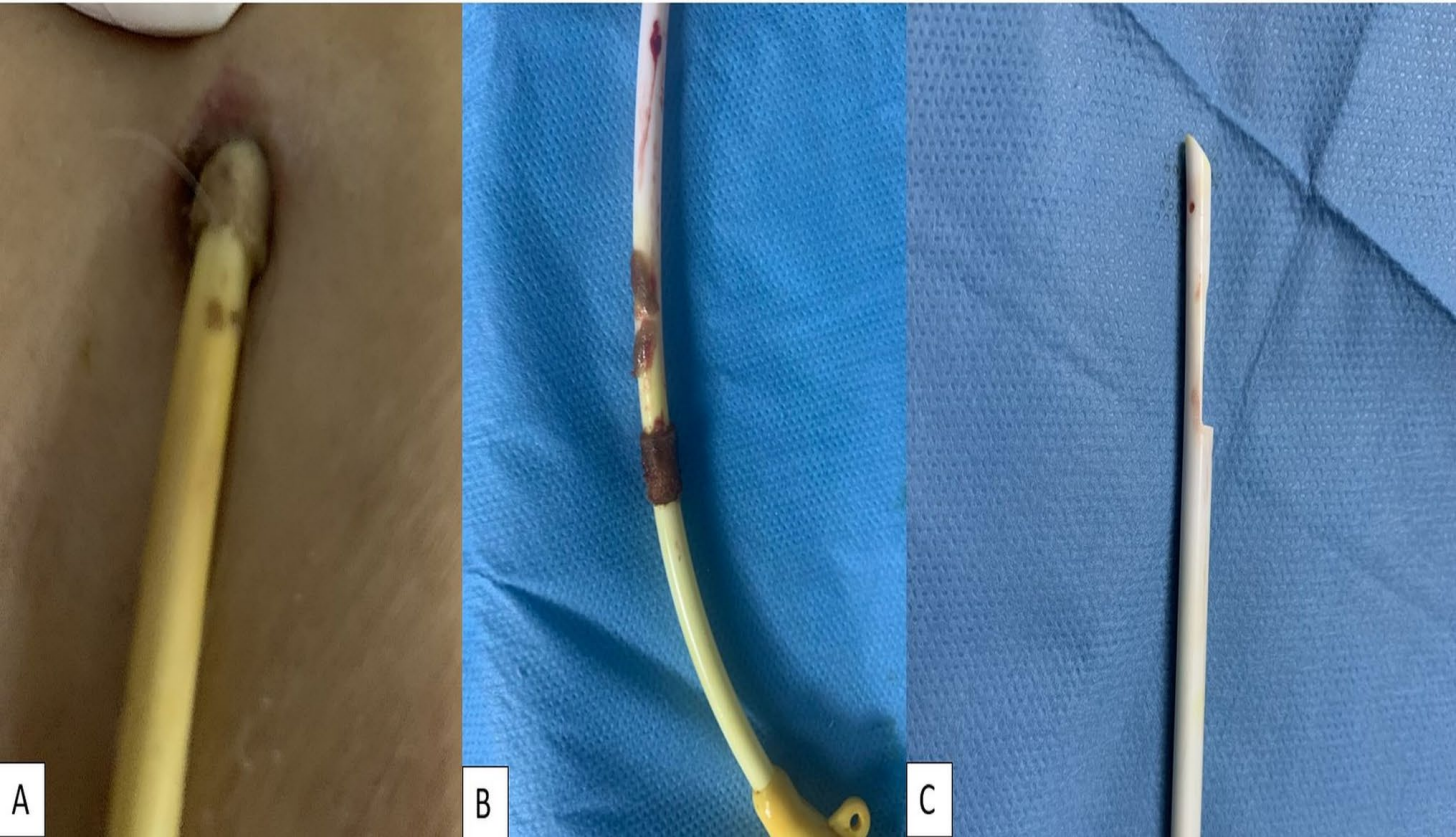
A patient with catheter-related infection displaying exit-site/tunnel infection (**A**). An exchange procedure was performed on the infected catheter showing biofilm on the surface (**B**), and subsequently, the tip (**C**) was sent for microbiology testing

The study cohort was initially categorized into two groups: (i) individuals admitted for the initial placement of tunneled hemodialysis catheters and (ii) those admitted for catheter exchange. Subsequently, the exchange group was further subdivided into four subgroups based on the reasons for the procedure: infection, dysfunction, displacement, and transition from temporary hemodialysis catheters to long-term ones (Graphic 1). Catheter-related infection refers to any infection affecting the vascular access (intraluminal/access, extraluminal/access, peri-access), leading to significant clinical indications of infection [11] (Fig. 1). Catheter dysfunction encompasses complications related to both thrombotic flow and non-thrombotic flow issues. Stenosis, particularly associated with the risk or occurrence of thrombosis, diminishes intra-access flow, posing a threat to the necessary access patency required for prescribed dialysis and/or causing clinical signs and symptoms. Non-thrombotic flow-related dysfunctions such as arteriovenous access aneurysms and steal syndrome may or may not directly impede flow or patency but are linked with clinical signs and symptoms [11].

Patients with partial or complete detachment of the cuff were included in the catheter displacement group. The final group comprised patients admitted for the transition from temporary hemodialysis catheters to long-term catheters, specifically those without any reported complaints or complications.

### Technique

The tunneled dialysis catheters equipped with a dacron cuff (Hemo Flow ® double internal lumen, 24-28-32F, MedComp, 1499 Delp Drive, Harleysville, USA) were either inserted or replaced by interventional radiologists within the operating room, maintaining sterile conditions and utilizing fluoroscopic and ultrasonographic guidance. Consistent with universal guidelines and recommendations, the right internal jugular vein was the primary choice for catheter placement. However, in certain cases where patient anatomy or other factors were involved, alternative veins such as the left jugular, subclavian, and femoral veins were preferred for catheter insertion.

**Fig. 2:**
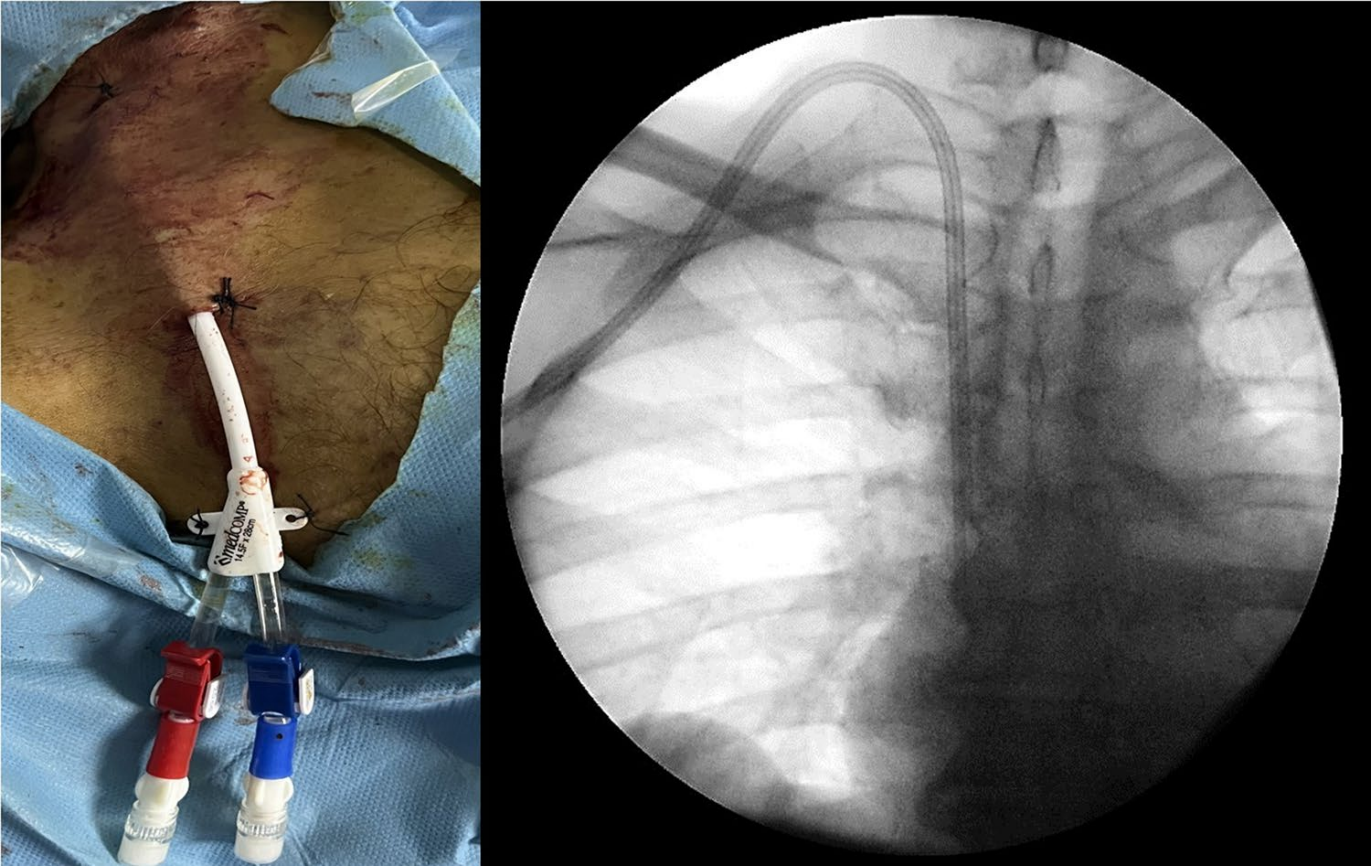
Placement of the initial catheter into the right jugular vein, followed by fluoroscopic imaging to confirm the catheter's proper positioning at the atrio-caval junction

At our institution, intravenous cefazolin (1 g) was given as antibiotic prophylaxis 30 min prior to the procedure. For individuals with penicillin allergy, clindamycin 600 mg IV was administered. Following the application of local anesthesia, the vein was accessed using an 18 G needle under sonographic guidance. A 0.035-inch wire was then inserted through the needle and guided to the inferior vena cava with the assistance of fluoroscopy. Measurements were taken to determine the appropriate tunnel size, and local anesthetic was administered subcutaneously from the chest wall insertion site toward the venotomy site. Subsequently, three dilators of varying sizes were employed to dilate the path around the guide wire, enabling easy passage of the multi-lumen catheter into the vein. A peel-away sheath was then placed over the wire, allowing for the insertion of the catheter through this sheath. Both lumens of the catheter were examined and heparinized as per catheter protocol. Suturing was performed at the venotomy site and catheter insertion site to secure the position (Fig. 2). Standard hemodialysis was administered to all patients.

### Statistical analysis

Statistical analyses were performed with the SPSS version 25.0. The conformity of the variables to the normal distribution was examined by histogram graphics and the Kolmogorov–Smirnov test. Mean, standard deviation, and median values were used when presenting descriptive analyses. The Mann–Whitney *U* test was used when evaluating nonparametric variables between two groups. The factors affecting the catheter change and its causes were examined by Binary Logistic Regression Analysis. Results with a p-value below 0.05 were considered statistically significant.

## Results

### Demographic data

The study comprised 1279 procedures for tunneled hemodialysis catheter placement conducted on 902 patients. Among these patients, 464 (51.4%) were female and 438 (48.5%) were male. The mean age of the patients was 63.5 with a standard deviation of 14.6.

Patient characteristics related to their history of catheter initial placement and exchange procedures are summarized in Graphic 2.

Descriptive analyses of platelet, neutrophil, lymphocyte, and monocyte counts and mean platelet volume, neutrophil–lymphocyte ratio, lymphocyte-monocyte ratio, and platelet-lymphocyte ratio values are given in Table 1.

**Table 1:** Descriptive analysis of platelet, neutrophil, lymphocyte and monocyte counts and MPV, NLR, LMR, PLR values

	Median (Q1–Q3)
PLT	230.00 (33.0–837.0)
NEUT	5.66 (1.0–39.57)
LYMPH	1.35 (0.08–5.96)
MONO	0.68 (0.03–2.88)
MPV	10.20 (7.30–13.90)
NLR	4.17 (1.0–44.61)
LMR	2.06 (0.32–8.77)
PLR	166.29 (31.53–936.67)

*PLT* Platelet, *NEUT* Neutrophil, *LYMPH* Lymphocyte, *MONO* Monocyte, *MPV* Mean platelet volume, *NLR* Neutrophil-to-lymphocyte ratio, *LMR* Lymphocyte-to-monocyte ratio, *PLR* Platelet-to-lymphocyte ratio

### Comparison of findings according to procedure type

In the exchange group, lymphocyte and monocyte values were found to be significantly higher (*p* < 0.001) whereas neutrophil–lymphocyte ratio and platelet-lymphocyte ratio values were lower (*p* < 0.001), (Table 2).

**Table 2:** Descriptive and statistical analysis of hemogram parameters according to procedure type

	Initial placement	Exchange	*p*
	Median (Q1–Q3)	Median (Q1–Q3)	
PLT	225.50 (79–837)	233.00 (33–717)	0.507
NEUT	5.66 (1.97–39.57)	5.66 (1–32.22)	0.290
LYMPH	1.21 (0.23–5.63)	1.41 (0.08- 5.96)	** < 0.001**
MONO	0.65 (0.08–2.25)	0.70 (0.03–2.88)	** < 0.001**
MPV	10.20 (7.3–13.9)	10.20 (7.8–13.9)	0.416
NLR	4.84 (1.03–44.61)	4.00 (1–39.71)	** < 0.001**
LMR	2.02 (0.32–8.75)	2.08 (0.43–8.77)	0.154
PLR	179.92 (47.78–936.67)	161.03 (31.53–761.7)	** < 0.001**

A *p*-value of 0.05 or lower is considered statistically significant (in bold)

*PLT* Platelet, *NEUT* Neutrophil, *LYMPH* Lymphocyte, *MONO* Monocyte, *MPV* Mean platelet volume, *NLR* Neutrophil-to-lymphocyte ratio, *LMR* Lymphocyte-to-monocyte ratio, *PLR* Platelet-to-lymphocyte ratio

Furthermore, a one-unit increase in monocyte count correlates with a 2.15 times higher rate of exchange. Conversely, a one-unit increase in neutrophil–lymphocyte ratio and platelet-lymphocyte ratio corresponds to a decrease in the exchange rate by 0.95 and 0.99 times, respectively (*p* < 0.05).

### Comparison of indications for exchange and blood count ratios

The subgroup analysis within the exchange group revealed that patients in the transition group exhibited higher monocyte values and lower mean platelet volume and lymphocyte-monocyte ratio values (*p* < 0.05). In the subgroup of infection; values for neutrophil count, mean platelet volume, neutrophil–lymphocyte ratio, and platelet-lymphocyte ratio were significantly higher compared to other groups (*p* < 0.05). A one-unit increase in mean platelet volume results in a 1.379 times increase in infection-related exchanges. Moreover, a one-unit rise in the neutrophil–lymphocyte ratio corresponds to a 1.078-fold increase in the likelihood of infection-related exchanges. Catheter dysfunction cases showed higher lymphocyte-monocyte ratio values and lower neutrophil, monocyte, neutrophil–lymphocyte ratio, and platelet-lymphocyte ratio values (*p* < 0.05).

Subgroup analysis of catheter exchange patients is summarized in Table 3 and Graphic 3.

**Table 3:** Descriptive and statistical analysis of hemogram parameters in subgroups of exchange procedure

	No	Yes	*p*
	Median (Q1-Q3)	Median (Q1-Q3)	
**Transition**			
PLT	228 (33–717)	239 (46–563)	0.053
NEUT	5.56 (1.6–32.22)	5.8 (1–27.4)	0.168
LYMPH	1.41 (0.08–5.96)	1.4 (0.29–4.79)	0.830
MONO	0.69 (0.03–2.88)	0.72 (0.04–2.72)	**0.034**
MPV	10.2 (8–13.6)	10.1 (7.8–13.9)	**0.045**
NLR	3.89 (1–31.98)	4.18 (1.04–39.71)	0.339
LMR	2.17 (0.43–8.77)	1.96 (0.46–8.55)	**0.021**
PLR	155.29 (34.52–761.7)	168.62 (31.53–637.78)	0.051
**Infection**			
PLT	234 (46–717)	220.5 (33–601)	0.391
NEUT	5.6 (1–27.4)	6.4 (1.6–32.22)	**0.012**
LYMPH	1.42 (0.29–4.79)	1.25 (0.08–5.96)	**0.005**
MONO	0.69 (0.04–2.72)	0.73 (0.03–2.88)	0.107
MPV	10.2 (7.8–13.9)	10.5 (8–13.2)	**0.002**
NLR	3.91 (1–39.71)	4.78 (1.54–31.98)	** < 0.001**
LMR	2.13 (0.43–8.55)	1.73 (0.47–8.77)	** < 0.001**
P LR	158.54 (31.53–754.55)	183.98 (34.52–761.7)	**0.044**
**Dysfunction**			
PLT	235 (33–601)	229 (57–717)	0.286
NEUT	5.89 (1–32.22)	5.45 (1.87–20.94)	**0.003**
LYMPH	1.38 (0.08–5.96)	1.46 (0.39–4.38)	0.087
MONO	0.72 (0.03–2.88)	0.67 (0.15–2.04)	**0.001**
MPV	10.2 (7.8–13.9)	10.2 (8–13.6)	0.711
NLR	4.33 (1.04–39.71)	3.6 (1–31.38)	** < 0.001**
LMR	1.95 (0.46–8.77)	2.3 (0.43–6.66)	** < 0.001**
PLR	169.12 (31.53–761.7)	150.71 (36.97–754.55)	**0.004**
**Displacement**			
PLT	233 (33–717)	228.5 (123–343)	0.485
NEUT	5.66 (1–32.22)	5.98 (2.28–22.64)	0.812
LYMPH	1.41 (0.08–5.96)	1.49 (0.45–3.16)	0.420
MONO	0.69 (0.03–2.88)	0.72 (0.37–1.33)	0.725
MPV	10.2 (7.8–13.9)	10.1 (8–12.1)	0.363
NLR	3.99 (1–39.71)	4.25 (1.29–20.02)	0.707
LMR	2.08 (0.43–8.77)	2.29 (0.57–4.22)	0.588
PLR	161.9 (31.53–761.7)	150.07 (70.88–497.83)	0.331

A *p*-value of 0.05 or lower is considered statistically significant (in bold)

*PLT* Platelet, *NEUT* Neutrophil, *LYMPH* Lymphocyte, *MONO* Monocyte, *MPV* Mean platelet volume, *NLR* Neutrophil-to-lymphocyte ratio, *LMR* Lymphocyte-to-monocyte ratio, *PLR* Platelet-to-lymphocyte ratio

## Discussion

Various inflammatory markers have been recently recognized as having different levels of prognostic significance in chronic illnesses, including malignancies [12].

Studies in the literature have explored inflammatory markers like the neutrophil–lymphocyte ratio and platelet-lymphocyte ratio, examining their predictive value in both chronic kidney disease patients undergoing dialysis and those not on dialysis [13, 14]. However, despite searching through the main medical databases such as PubMed, Embase, Google Scholar, Scopus, and Web of Science, comprehensive studies exploring the predictive potential of systemic inflammatory markers on the survival of tunneled catheters, particularly with such a large sample size, have not yet been conducted. Our study represents a pioneering investigation into these parameters within subgroups of patients with different reasons for catheter exchange.

In a previous study, Coker et al. found that none of the assessed factors could predict tunneled hemodialysis catheter infection [3]. However, numerous other studies involving hemodialysis patients, though not specifically focusing on catheters, have highlighted the value of mean platelet volume, neutrophil–lymphocyte ratio, and platelet-lymphocyte ratio values in predicting mortality [15], risk of hospitalization [16], and inflammation [10, 17]. In our study, some markers were identified as significantly associated with the transition, infection, and dysfunction groups.

The neutrophil–lymphocyte ratio has been observed to rise alongside the inflammatory marker C-reactive protein in numerous studies [10]. Some research suggests that the neutrophil–lymphocyte ratio could serve as a readily accessible and cost-effective substitute for assessing interleukin-1 and tumor necrosis factor-alpha, which might not be available in every clinical setting [10, 18]. Catabay et al. demonstrated a negative correlation between the neutrophil–lymphocyte ratio and albumin levels, presenting the neutrophil–lymphocyte ratio as a robust predictor of all-cause mortality among hemodialysis patients. They emphasized that the predictive capability of the neutrophil–lymphocyte ratio surpasses that of the platelet-lymphocyte ratio [19].

In our study, both the mean (5.56 ± 4.79) and median (4.17) values of the neutrophil–lymphocyte ratio were higher than those commonly reported in hemodialysis patients [20, 21].

The group of patients in which the exchange of catheter was due to infection (infection-related catheter exchange group) exhibited significantly higher levels of the neutrophil–lymphocyte ratio compared to the other groups. These findings are consistent with the literature regarding the role of the neutrophil–lymphocyte ratio as an inflammatory marker. Indeed, various studies have proposed different cut-off values for the neutrophil–lymphocyte ratio as an inflammatory marker [15, 20, 22]. Jahangiri et al. reported that a neutrophil-lymphocyte ratio NLR > 5 holds significance in patients experiencing in-stent dysfunction [22]. Other researchers investigating catheter dysfunction in hemodialysis patients found that the values of neutrophil–lymphocyte ratio and platelet-lymphocyte ratio lacked statistical significance in predicting complications, suggesting they may serve only as supplementary markers [3, 16]. Due to low sensitivity and specificity, we were unable to establish any definitive cut-off value. The clinical significance of the neutrophil–lymphocyte ratio when used alone remains uncertain. We suggest its use alongside other clinical data to predict catheter dysfunction. Additionally, the parameters mentioned above are elevated in various conditions in HD patients, making it challenging to pinpoint the cause of these elevations [6–8, 21]. To address this, our study excluded patients with malignancies and performed hemogram measurements within 24 h prior to the procedure.

The mean (10.27) and median (10.2) values of mean platelet volume among our patients align with similar characteristics observed in other hemodialysis populations [23, 24]. Notably, in the infection-related catheter exchange group, the mean platelet volume was higher compared to both the initial placement group (*p* = 0.045) and the transition group (*p* = 0.009). Gao et al. reported that mean platelet volume may serve as a prognostic indicator in patients with septic shock. Their study revealed that mean platelet volume ranked second only to lactate in terms of the area under the curve (0.81), boasting a precision rate of 75.6% at a cut-off of 10 [23]. Another independent study established mean platelet volume as a robust predictor of fatal outcomes in secondary sepsis [25]. Elevated mean platelet volume signifies increased platelet activity, faster aggregation, and increased release of inflammatory cytokines [25, 26]. This phenomenon contributes to intimal hyperplasia, expediting atheroma plaque formation, and augments the thrombotic risk associated with platelet activation [27]. Lano et al.’s study on hemodialysis patients supported these findings by highlighting that individuals with the highest mean platelet volume faced heightened risks of vascular access complications, such as stenosis and thrombosis [24].

However, in our study, we did not observe a statistically significant difference in thrombosis and stenosis complications according to mean platelet volume (*p* = 0.062).

Several studies on hemodialysis patients have discussed the predictive role of the platelet-lymphocyte ratio, but none specifically addressed its relevance to catheter survival. Yaprak et al. concluded that the platelet-lymphocyte ratio surpasses the neutrophil–lymphocyte ratio in predicting mortality [15]. Similarly, Muresan et al. identified the platelet-lymphocyte ratio, along with the neutrophil–lymphocyte ratio and lymphocyte-monocyte ratio, as independent predictors of averse outcomes in chronic kidney disease patients [28].

In our study, we observed a lower platelet-lymphocyte ratio in the group patients neededing catheter exchange compared to individuals with initial catheter placement. However, upon subgroup analysis, we found a significantly higher platelet-lymphocyte ratio in the infection-related exchange group. Given that catheter infection poses a major risk for morbidity and mortality, our findings align with existing literature highlighting the prognostic value of the platelet-lymphocyte ratio.

When comparing our research outcomes with existing studies, the neutrophil–lymphocyte ratio and platelet-lymphocyte ratio emerge as significant factors in both infection and dysfunction groups, exhibiting a stronger correlation in the infection subgroup. Conversely, the lymphocyte-monocyte ratio shows greater correlation in the dysfunction group. While lymphocyte-monocyte ratio has been explored in various medical contexts, its specific predictive value in thrombosis might be under-researched. Studies examining inflammatory markers, including lymphocytes and monocytes, indirectly relate to thrombosis, shedding light on the association between these cell ratios and thrombotic risks [29, 30].

Overall, our study suggests that hemogram parameters play a crucial role in predicting catheter survival and their acknowledgement may influence clinical treatment decisions. However, these parameters require validation through prospective, randomized studies that include a control group and assess long-term catheter outcomes.

Our study's primary limitation lies in its retrospective nature, possibly leading to selection bias and confounding by indication. Future prospective studies are essential to validate these data.

Hemodialysis patients often present with comorbidities that might influence hemogram parameters. While we excluded specific comorbidities, other underlying diseases could not be entirely ruled out.

Accumulating disease-free/uncomplicated catheter-day data is crucial for analyzing catheter survival, yet obtaining such information was challenging due to the large patient population, incomplete medical histories, and inadequate follow-up [31].

This research was conducted within our interventional radiology clinic, working not only with  our hospital hemodialysis patients but also with external clinics. Limited interaction with external clinics was a drawback. Addressing this limitation in prospective studies with enhanced collaboration with nephrologists is imperative.

In conclusion, several parameters including monocyte counts, neutrophil–lymphocyte ratio, mean platelet volume, lymphocyte-monocyte ratio, and platelet-lymphocyte ratio stand as valuable supportive markers aiding in decision-making in tunneled catheter procedures. The establishment of definitive cut-off values to shape an algorithm remains an unmet goal to be pursued in the future.

### Supplementary Information

Below is the link to the electronic supplementary material.

Supplementary file1 (TIF 111 KB)

Supplementary file2 (TIF 301 KB)

Supplementary file3 (TIF 133 KB)

## Data Availability

The datasets used and analyzed during the current study are available from the corresponding author on reasonable request.
