# Supplementary figures and images for: The predictive value of systemic inflammatory markers in 902 patients with tunneled hemodialysis catheter
Source: J Nephrol. 2024 Mar 21;37(4):1041–9. doi: 10.1007/s40620-023-01880-w (PMC11239775; doi:10.1007/s40620-023-01880-w)

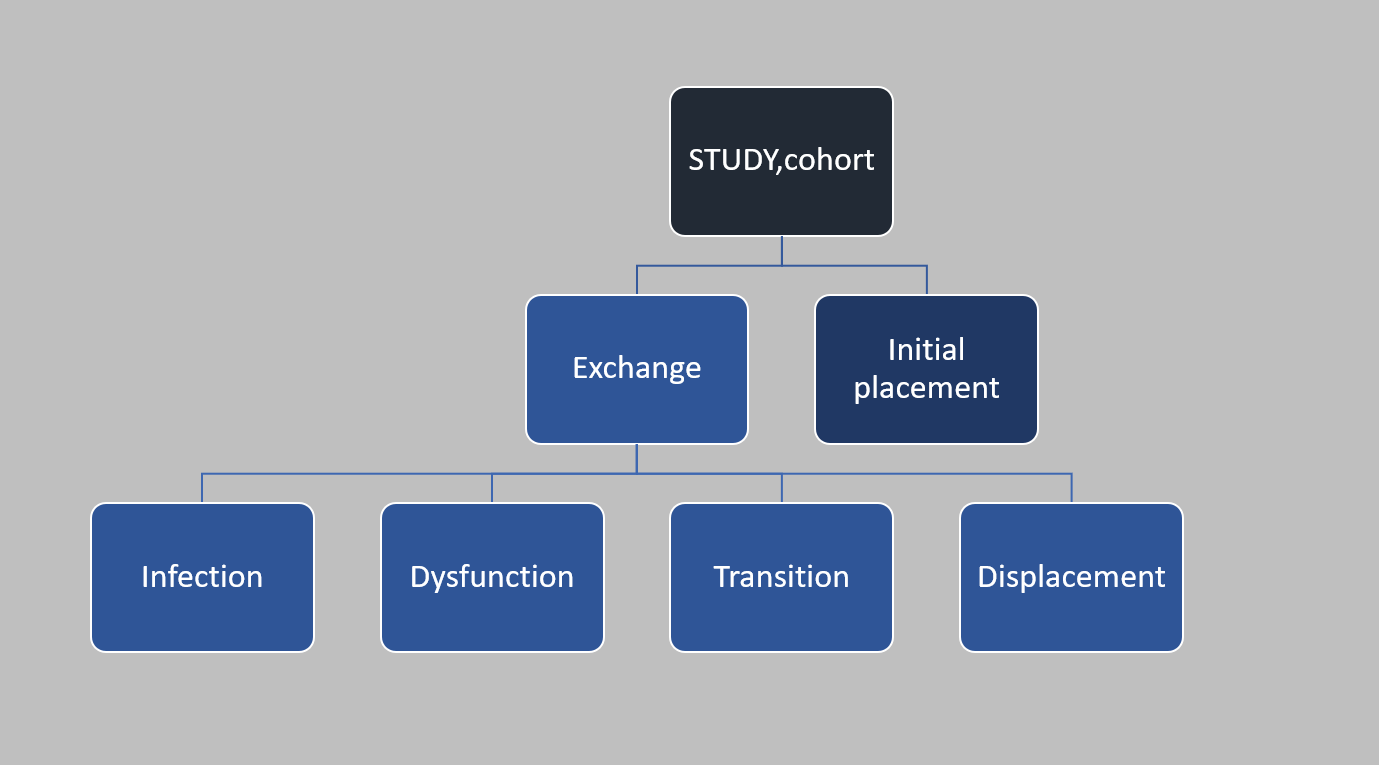

Supplement: Supplementary file 1 — Supplementary file1 (TIF 111 KB) [file 40620_2023_1880_MOESM1_ESM.tif]

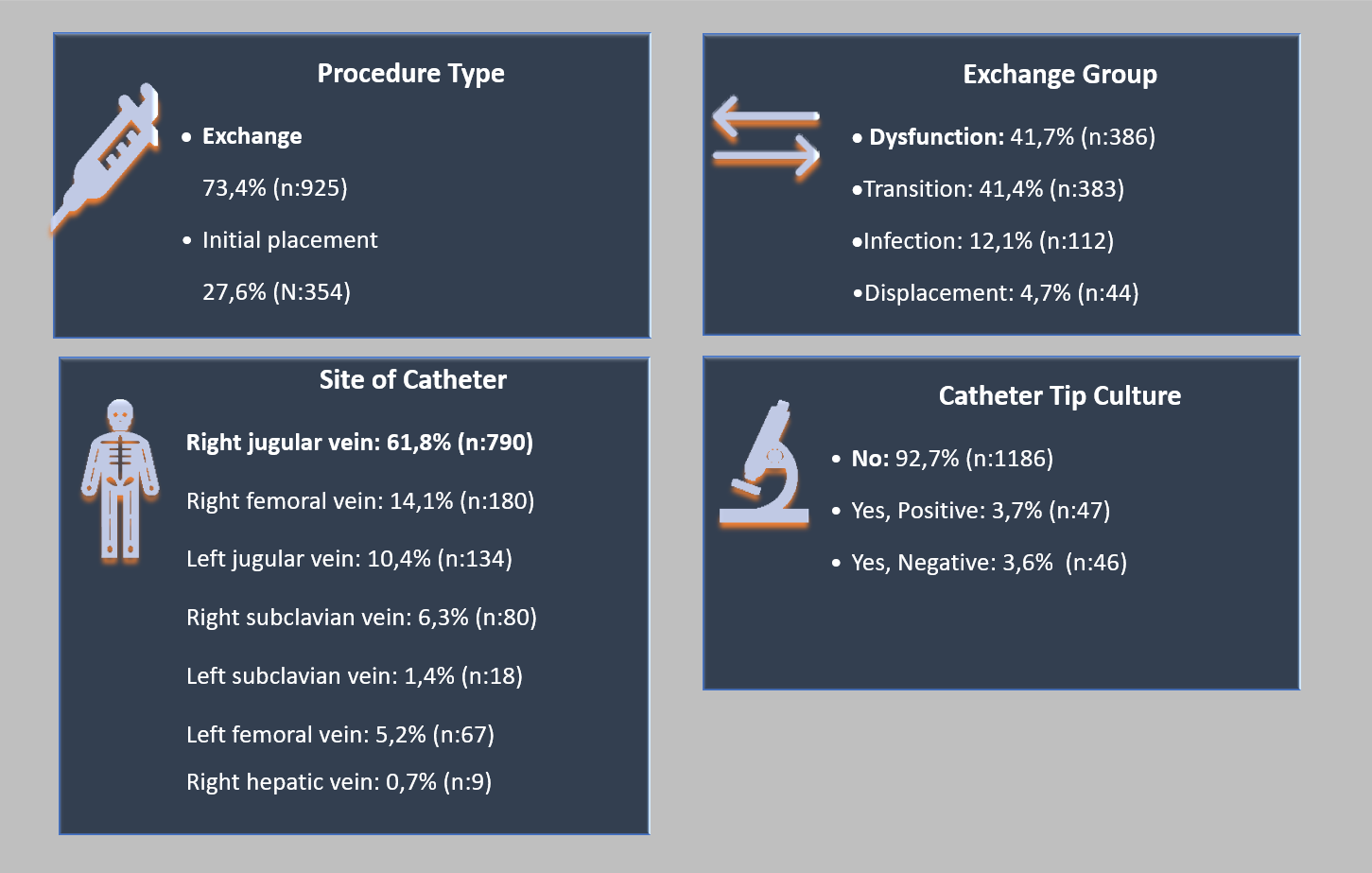

Supplement: Supplementary file 2 — Supplementary file2 (TIF 301 KB) [file 40620_2023_1880_MOESM2_ESM.tif]

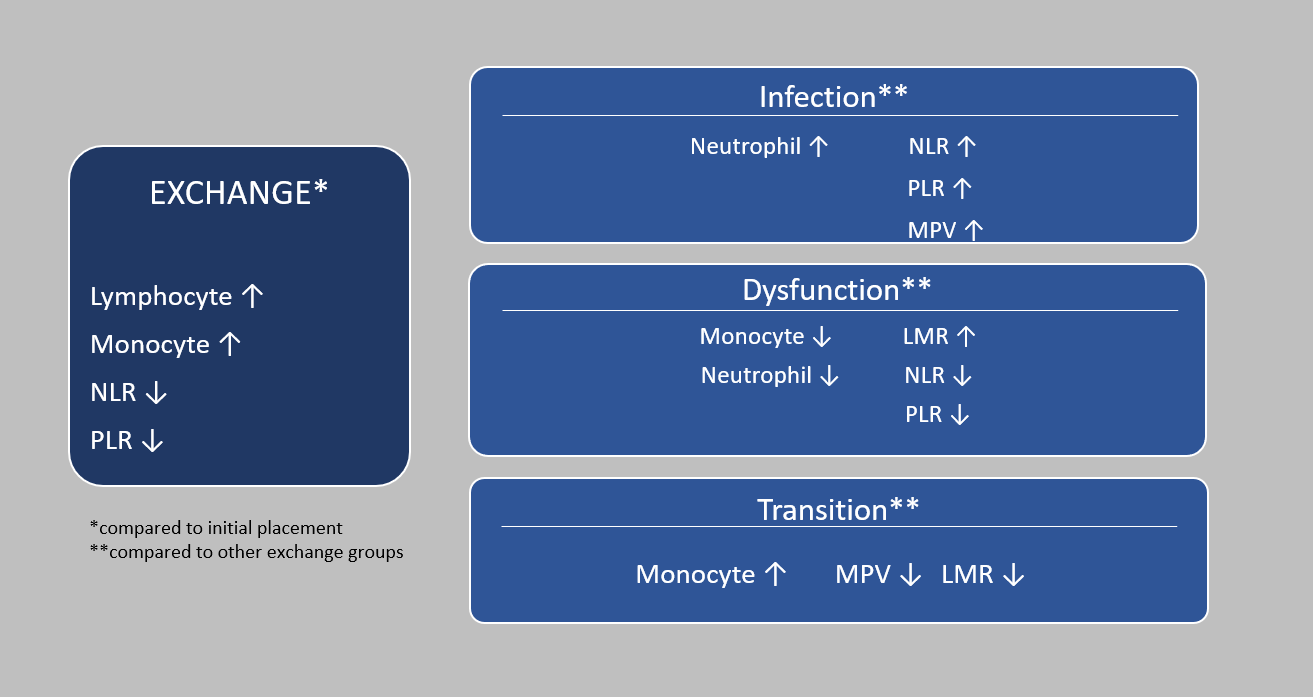

Supplement: Supplementary file 3 — Supplementary file3 (TIF 133 KB) [file 40620_2023_1880_MOESM3_ESM.tif]
